# The role of vascular endothelial growth factor as a prognostic and clinicopathological marker in osteosarcoma: a systematic review and meta-analysis

**DOI:** 10.1186/s13018-021-02888-3

**Published:** 2021-12-28

**Authors:** Chao Zhang, Lin Wang, Chuang Xiong, Runhan Zhao, Hao Liang, Xiaoji Luo

**Affiliations:** 1 Department of Orthopedics, The First Affiliated Hospital of Chongqing Medical University, Chongqing, 400016 People’s Republic of China; 2 Orthopedic Laboratory of Chongqing Medical University, Chongqing, People’s Republic of China

**Keywords:** Vascular endothelial growth factor, Osteosarcoma, Prognosis, Meta-analysis

## Abstract

**Background:**

In recent years, numerous investigations have been conducted to determine the clinical significance and critical functions of vascular endothelial growth factor (VEGF) in various malignant cancers. The purpose of this meta-analysis was to comprehensively evaluate the prognostic and clinicopathological value of VEGF in patients with osteosarcoma.

**Methods:**

We performed a systematic literature retrieval of available databases. Odds ratios (ORs) or standard mean difference (SMD) for clinicopathological parameters, hazard ratios (HRs) for overall survival and disease-free survival were calculated to assess the correlation between VEGF expression and prognosis in patients with osteosarcoma.

**Results:**

A total of 22 studies with 1144 patients were included in our study. Pooled analyses showed that VEGF overexpression predicted worse overall survival (HR, 2.42; 95% CI, 1.87–3.11, *p* < 0.001) and disease-free survival (HR, 2.604; 95% CI, 1.698–3.995, *p* < 0.001), respectively. Furthermore, investigation regarding osteosarcoma clinicopathologic characteristics suggested that high VEGF expression was significantly associated with metastasis (OR, 4.39; 95% CI, 2.77–6.95; *p* < 0.001), clinical stage (OR, 0.73; 95% CI, 0.62–0.87; *p* < 0.001), and microvessel density (SMD, 3.33, 95% CI,1.57–5.10, *p* < 0.001), but not associated with tumor location, gender, age, local recurrence, and chemotherapy response.

**Conclusion:**

Our meta-analysis findings suggest that elevated VEGF expression may be a predictive biomarker for poor prognosis and adverse clinicopathological characteristics in patients with osteosarcoma.

## Introduction

Osteosarcoma is the most frequent malignant osteogenic tumor, mostly occurring in children and young adults [1]. Over the past decade, the clinic appliance of neoadjuvant reduced the size of the localized tumor and delayed the progression, significantly improving the 5-year survival rate of patients with low-grade osteosarcoma [2]. However, metastasis has been reported to be present in approximately 25% of newly diagnosed osteosarcoma patients, and the mortality rate in these patients remains extremely high at approximately 20% [2–4]. There is currently an absence of viable methods for the early diagnosis and treatment of osteosarcoma. Given this, further investigation of prognostic molecular biomarkers is critical for a better understanding of osteosarcoma's pathophysiology and the development of more effective treatment modalities.

Angiogenesis plays a vital role in tumor development as the growth of tumors relies on the perfusion of neovascular [5]. Vascular endothelial growth factor (VEGF) is a potent pro-angiogenic factor that regulates vascular endothelial cell proliferation, differentiation and migration [6]. Overexpression of VEGF has been reported to be attributed to the invasion and metastasis of a wide range of solid tumors [7–9]. To clarify the mechanism of VEGF in the advancement of osteosarcoma, the association between VEGF and prognosis features of osteosarcoma has been assessed. However, the prognostic and clinicopathological value of VEGF remains controversial [10, 11]. Previous relevant meta-analyses have been performed to define the clinical significance of VEGF expression in osteosarcoma. Nevertheless, these analyses were inconclusive as inconsistent results, limited involved studies, and the absence of a thorough evaluation of study quality and pooled results [12–14]. Therefore, this current study aimed to comprehensively and systematically assess the prognostic value of VEGF in 22 studies involving 1144 osteosarcoma patients.

## Materials and methods

This study was conducted entirely in accordance with the Preferred Reporting Items for Systematic Reviews and Meta-Analyses (PRISMA) [15].

### Search strategies

A comprehensive electronic literature search was performed in four databases: Web of Science, PubMed, Cochrane Library and Medline with no restrictions on language or publication date. The last search was conducted on September 12, 2021. The following terms were used to conduct the literature search: ("osteosarcoma" or "osteogenic sarcoma") and ("vascular endothelial growth factor" or VEGF). We additionally manually screened the references of identified articles to collect more studies.

### Selection criteria

The eligible articles were selected in accordance with the following criteria: (1) patients were diagnosed with osteosarcoma pathologically; (2) the relationship between VEGF expression and clinicopathological characteristics or prognosis were investigated; (3) the expression VEGF was determined on samples of tumor tissue. Articles were excluded according to the following criteria: (1) studies were published in the form of conference abstracts, letters, case reports, expert opinions, reviews, or sequence data; (2) focused on tumor cell lines or animal experiments; (3) patients did not confirm the diagnosis of osteosarcoma; (4) when study comprised overlapping patient cohort. Two independent authors determined whether studies were eligible. Any discrepancies were settled by consensus following a discussion.

### Data extraction and quality assessment

Two independent investigators carefully reviewed all eligible publications to extract interested data. The following data were collected, including (1) first author, publication year, patient source; (2) number of patients, age, gender, VEGF assay method, antibody type, source and dilution of immunohistochemistry (IHC), and cutoff value; (3) tumor stage at diagnosis, metastasis, local recurrence, tumor location, chemotherapy response, microvessel intensity (MVD), hazard ratio (HR) of VEGF expression and corresponding 95% CI.

If a study stated both univariate and multivariate survival results, the HRs from the multivariate analyses were used. When the survival results were not given explicitly while a Kaplan–Meier curve was present, the HRs with 95% CIs were retrieved using Engauge Digitizer 11.0 software and Tierney’s reported method [16].

Each involved study’s quality was assessed using the Newcastle–Ottawa Scale (NOS) by two independent reviewers [17]. The scale judges the quality of studies from three main aspects: the selection of the groups, comparability, and exposure, with a maximum of nine points. Articles with a NOS score of more than six were considered to be of high quality.

### Statistical analysis

The statistical analysis in this study was performed by STATA 14.0 (Stata Corporation, College Station, TX, USA). We estimate the pooled HRs for survival results, the pooled odds ratio (OR) for the clinicopathological characteristics (age, gender, stage, metastasis, local recurrence, response to chemotherapy). The continuous variables are described as standard mean difference (SMD). The statistical between-study heterogeneity was assessed by the Chi-squared test and the Higgins *I*^2^ statistic. Significant heterogeneity was defined as a *p* > 0.10 or *I*^2^ > 50%. A fixed-effects model was utilized when there was no significant heterogeneity. Otherwise, a random-effects model was utilized. The potential publication bias was estimated by using Begg’s funnel plot and Egger’s test. Additionally, we performed sensitivity analyses to assess the stability of the pooled outcomes. *p* < 0.05 was considered statistically significant.

## Results

### Search results

**Fig. 1:**
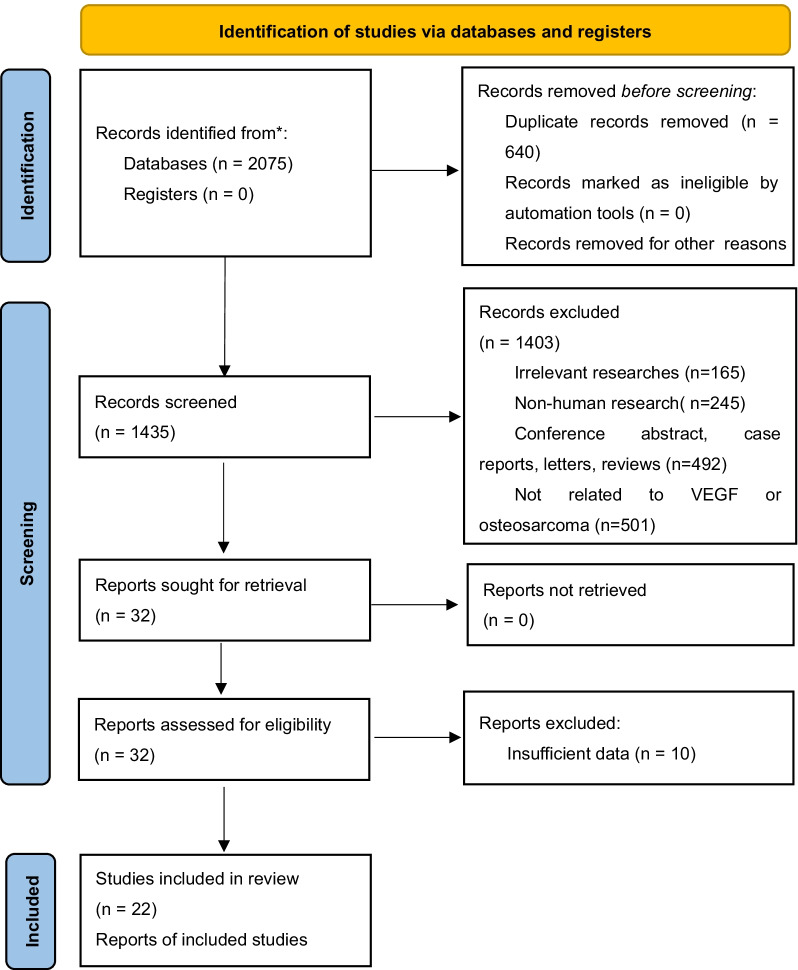
The flowchart of the study selection in this meta-analysis

A total of 2075 articles were identified from four online databases. After removing 640 duplicates, the remaining 1435 records were systematically evaluated by the titles and abstracts. Among these articles, 165 articles were excluded for irrelevant studies, 245 articles involved non-human experiments, 492 articles were conference abstracts, case reports, letters, and reviews, and 501 articles were not related to VEGF or osteosarcoma. After assessing the entire text of the remaining 32 studies, 10 articles were excluded for insufficient data. Finally, 22 studies with a total of 1144 osteosarcoma patients were included in this study [10, 11, 18–37]. The detailed flowchart of the study filtrating process is shown in Fig. 1.

### Study characteristics and quality assessment

The summarized characteristics of the included study are shown in Table 1. Among them, 11 studies focused on the prognostic significance, 22 studies analyzed the correlation between VEGF expression and clinicopathological characteristics. All of the eligible research was published between 1999 and 2020, and it was written in English, with a patient population ranging from 25 to 153. Additionally, immunochemical staining (IHC) was the most often employed technique to measure VEGF expression (21/22, 95.5%), with 95.2% of the studies using IHC having defined the cutoff value of VEGF expression. Each article used the tissue as the sample. In terms of study quality, all of the eligible studies were high quality with a NOS score greater than 6 points. Other information about the involved studies is shown in Table 1.

**Table 1:** Main characteristics of the studies included in this meta-analysis

Study	Year	Patient source	Antibody type	Antibody dilution	Number of patients	Tumor stage	Method and isoforms	Cutoff value	HR Source	Outcome	NOS score
Kong	2020	China	Beijing Bioss Biotech	1:100	37	I, II, III	IHC	≥ 2^a^	SC	OS, CPF	6
Mohamed	2019	Egypt	Santa Cruz	1:100	66	II, III	IHC	> 30%^b^	NA	CPF	6
Wu	2019	China	Santa Cruz	1:100	53	I, II, III	IHC	≥ 4^a^	COX	OS, CPF	7
Liu	2017	China	Santa Cruz	NA	84	I, II, III	IHC	≥ 1^a^	COX	OS, CPF	7
Lei	2015	China	Abcam	1:150	32	I, II, III	IHC	> 3^a^	NA	CPF	6
Zhao	2015	China	NA	NA	153	I, II, III	IHC	≥ 4^a^	SC	OS, CPF	8
Baptista	2014	Brazil	Dako	1:100	50	I, IIA, IIB	IHC	> 30%^b^	SC	OS, DFS, CPF	8
Becker	2013	Brazil	Dako	1:50	27	IIB III	IHC	> 30%^b^	NA	CPF	6
Lammli	2012	USA	Santa Cruz	NA	54	NA	IHC	≥ 20%^b^	NA	CPF	6
Chen	2012	China	Santa Cruz	1:200	49	IIA, IIB, III	IHC	NA	SC	DFS, CPF	9
Zhou	2011	China	Santa Cruz	1:200	65	IIA, IIB, III	IHC	≥ 10%^b^	NA	CPF	6
Lin	2011	China	FuZhou JingXing Corporation	NA	56	II III	IHC	≥ 10%^b^	SC	OS, CPF	8
Lugowska	2011	Poland	Santa Cruz	4:2000	91	IIB, III	IHC	> 50%^b^	COX	OS, CPF	8
Abdeen	2009	USA	NA	NA	48	IIB, III	IHC	≥ 2points^c^	NA	CPF	7
Mizobuchi	2008	USA	Santa Cruz	1:200	48	NA	IHC	≥ 1, intensity of staining	NA	CPF	6
Huang	2008	China	Santa Cruz	1:100	31	I, IIA, IIB	IHC	≥ 1, intensity of staining	NA	CPF	6
Park	2008	Korea	Zymed Lab	1:100	35	NA	IHC	> 30%^b^	NA	CPF	6
Charity	2006	England	BD Biosciences Pharmingen	NA	53	I, II, III	IHC	≥ 25%^b^	COX	OS, DFS, CPF	6
Oda	2006	Japan	Santa Cruz	1:500	30	NA	IHC	IRS ≥ 2 + with focal to diffuse distributions	SC	OS, CPF	8
Jung	2005	Korea	Santa Cruz	1:200	25	NA	IHC	> 2 + , number of new vessel	NA	CPF	7
Kaya	2000	Jpan	Santa Cruz	1:200	27	I, II, III	IHC	> 30% ^b^	SC	OS, DFS, CPF	9
Lee	1999	Japan	–	–	30	NA	RT-PCR	–	SC	OS, CPF	6

HR, hazard ratio; IHC, immunohistochemistry; qRT-PCR: quantitative real-time polymerase chain reaction; NOS, Newcastle–Ottawa Scale; OS, overall survival; DFS, disease-free survival; SC, survival curve; IRS, immunoreactive score; NA, not available;

^a^The IRS was calculated by multiplication of percentage of stained cells and the intensity of staining;

^b^Total score was calculated by the number of positive cells;

^c^The staining was consistent with the control tissue and was of equal intensity to the positive control tissue

### VEGF expression and prognostic significance

The survival data, including overall survival (OS) or disease-free survival (DFS), were analyzed in 11 studies among eligible studies. Due to the lack of evident heterogeneity detected (*I*^2^ = 0.00%, *p* = 0.894), a fixed-effects model was utilized. The result showed that the elevated VEGF expression was associated with poor overall survival (HR, 2.42; 95% CI, 1.87–3.11, *p* < 0.001). To further find out the potential sources of heterogeneity, we undertook a subgroup analysis stratified by ethnicity, publication date, testing isoform, antibody type, positive rate, HR resource, sample size, and NOS score. As shown in Table 2, each subgroup presented a significant association with overall survival. Besides, disease-free survival was also extracted in studies. The fixed-effect model was employed to calculate the pooled HR (*I*^2^ = 0.00%, *p* = 0.485). Results reveal that the elevated VEGF expression predicted poor disease-free survival (HR, 2.604; 95% CI, 1.698–3.995, *p* < 0.001).

**Table 2:** The subgroups analysis for VEGF and overall survival in patients with osteosarcoma

Subgroup	Number of studies	Model	Heterogeneity test		Effect size			Conclusion
			*I*^2^	*p* value	HR	95%CI	*p* value	
*Ethnicity*								
Asian	8	Fixed	0.0%	0.774	2.49	1.85–3.35	< 0.001	Significant
Non-Asian	3	Fixed	0.0%	0.695	2.20	1.34–3.61	0.002	Significant
*Publication (year)*								
≥ 2014	5	Fixed	0.0%	0.833	1.94	1.32–2.83	0.001	Significant
< 2014	6	Fixed	0.0%	0.942	2.87	2.04–4.04	< 0.001	Significant
*Testing isoform*								
IHC	10	Fixed	0.0%	0.959	2.45	1.84–3.25	< 0.001	Significant
qRT-PCR	1	–	–	–	–	–	–	–
*Antibody type*								
Santa Cruz	5	Fixed	0.0%	0.896	2.65	1.83–3.83	0.001	Significant
Others	4	Fixed	0.0%	0.795	2.18	1.40–3.40	< 0.001	Significant
*Positivity (%)*								
≥ 55%	7	Fixed	0.0%	0.731	1.98	1.31–2.99	0.001	Significant
< 55%	4	Fixed	0.0%	0/892	2.71	1.96–2.75	< 0.001	Significant
*HR resource*								
Reported	5	Fixed	0.0%	0.868	2.20	1.87–3.11	< 0.001	Significant
SC	6	Fixed	0.0%	0.894	2.73	1.85–4.02	< 0.001	Significant
*Sample size*								
≥ 50	7	Fixed	0.0%	0.915	2.19	1.63–2.94	< 0.001	Significant
< 50	4	Fixed	0.0%	0.722	3.20	1.92–5.34	< 0.001	Significant
*NOS score*								
< 8	6	Fixed	0.0%	0.800	2.495	1.72–3.62	< 0.001	Significant
≥ 8	5	Fixed	0.0%	0.663	2.335	1.65–3.31	< 0.001	Significant

HR, hazard ratio; NOS, Newcastle–Ottawa Scale; VEGF, vascular endothelial growth factor; IHC, immunohistochemistry; qRT-PCR: quantitative real-time polymerase chain reaction

### VEGF expression and clinicopathological features

**Fig. 2:**
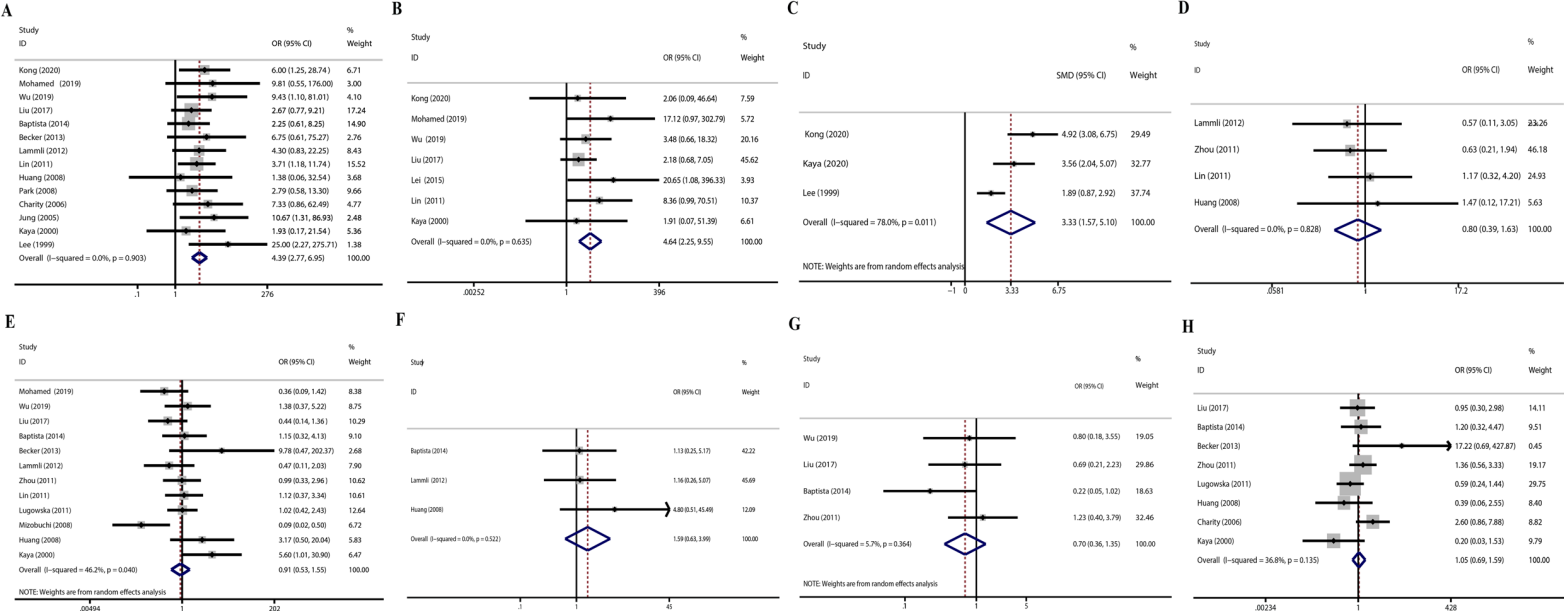
Forest plots of the association between VEGF overexpression and clinicopathological features. **A** Distant Metastasis. **B** Clinical stage. **C** Tumor location **D** MVD. **E** Gender. **F** Local recurrence. **G** Age. **H** Chemotherapy response. OR, odds ratio; CI, confidence intervals; MVD, microvessel density

The correlation between VEGF expression and clinicopathological values, including age, gender, metastasis, local recurrence, tumor stage, response to chemotherapy, and MVD, was investigated. A fixed-effects or a random-effects model was employed based on the heterogeneity results of each parameter. The detailed information is shown in Table 3. Under the fixed-effects model, overexpression of VEGF was significantly related to a higher rate of osteosarcoma metastasis (OR, 4.39; 95% CI, 2.77–6.95; *p* < 0.001). The random-effects model showed that the overexpression of VEGF was significantly related to a higher clinical stage (OR, 0.73; 95% CI, 0.62–0.87; *p* < 0.001). Besides, VEGF expression showed a significant correlation with microvessel density (MVD) according to the results of the random-effects model (SMD, 3.33, 95% CI,1.57–5.10, *p* < 0.001). However, we failed to find a significant relationship between overexpression of VEGF and gender, tumor location, local recurrence, age, and response to chemotherapy (Fig. 2).

**Table 3:** Pooled odds ratios of VEGF on clinicopathologic features in osteosarcoma

Variables	No. of studies	Heterogeneity test		Effect size			Model	Conclusion
		*I*^2^ (%)	*p* value	OR/SMD	95%CI	*p* value		
Distant Metastasis	14	0.0	0.903	4.39	2.77–6.95	< 0.001	Fixed	Significant
Clinical stage	7	0.0	0.635	0.22	2.25–9.55	< 0.001	Fixed	Significant
MVD	3	78.0	0.011	3.33	1.57–5.10	< 0.001	Random	Significant
Tumor location	4	0.0	0.828	0.799	0.39–1.63	0.538	Fixed	Not significant
Gender	12	46.2	0.040	0.91	0.53–1.55	0.726	Random	Not significant
Local recurrence	3	0.0	0.522	1.43	0.69–2.98	0.328	Fixed	Not significant
Age	4	5.7	0.290	0.71	0.37–1.34	0.364	Fixed	Not significant
Chemotherapy response	8	36.8	0.135	0.96	0.63–1.45	0.832	Fixed	Not significant

OR, odds ratio CI; confidence interval; SMD, standard mean difference; MVD, microvessel density; VEGF, vascular endothelial growth factor

### Publication bias and sensitivity analysis

**Fig. 3:**
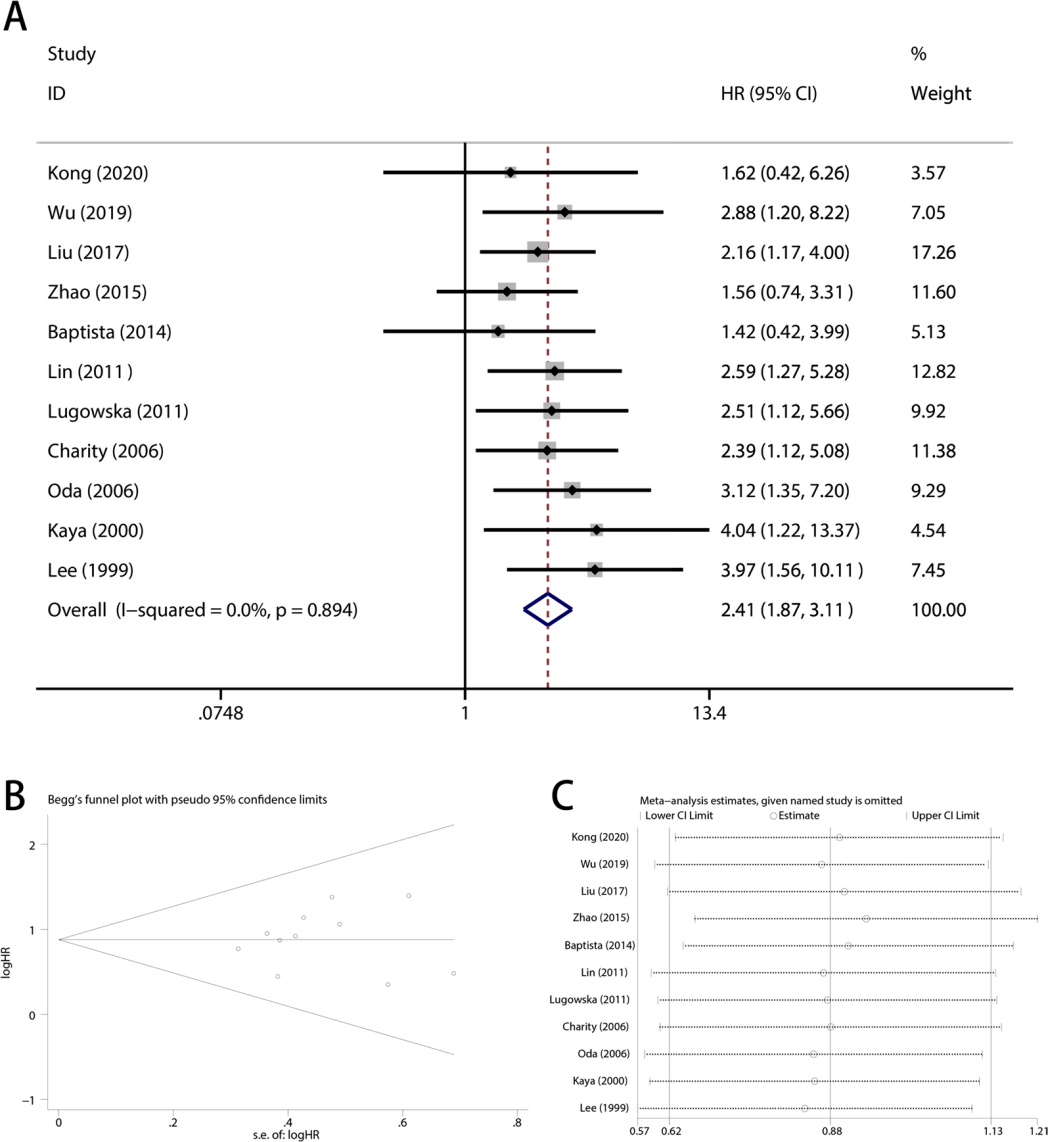
Pooled analysis for the association between VEGF overexpression and Overall survival. **A** Forest plots. **B** Funnel plots. **C** Sensitive analysis. OS, overall survival; OR, odds ratio; CI, confidence intervals; s.e., standard error

**Fig. 4:**
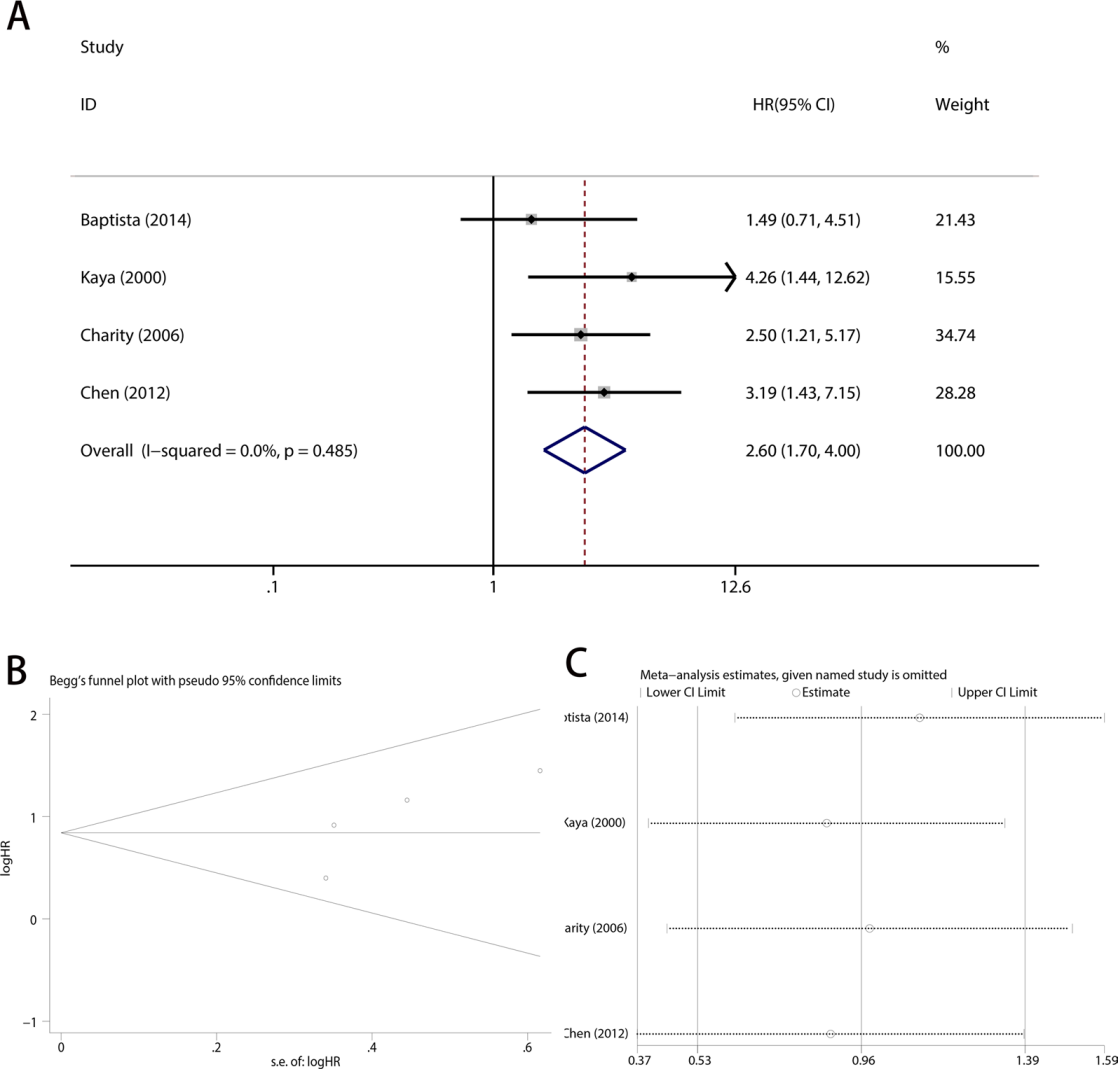
Pooled analysis for the association between VEGF overexpression and disease-free survival. **A** Forest plots. **B** Funnel plots. **C** Sensitive analysis. OS, overall survival; OR, odds ratio; CI, confidence intervals; s.e., standard error

Publication bias was measured by using Begg's funnel plot and Egger's tests. As shown in Figs. 3B and 4B, there was no publication bias for overall survival (Begg's test, *p* = 0.436; and Egger's test, *p* = 0.745) and disease-free survival (Begg's test, *p* = 0.089; and Egger's test, *p* = 0.198).

We performed a sensitivity analysis of overall survival and disease-free survival to investigate the influence of each study on the pooled HR. As Figs. 3C and 4C show, we did not find any significant alteration in the pooled HR when omitting any single study sequentially, demonstrating that the analyses were stable and credible.

## Discussion

As the most frequent primary osteogenic tumor, osteosarcoma is characterized as aggressive cancer with a high risk of distant metastasis. Although immune checkpoint inhibitors have recently revolutionized the treatment of a wide range of solid malignancies, they have demonstrated limited efficacy in osteosarcoma [38–40]. Therefore, the identification of other biomarkers related to the prognosis of osteosarcoma is crucially essential to the development of new potential therapeutic targets.

Angiogenesis is essential for the proliferation and metastasis of tumor cells [5]. In the past decades, VEGF has been the most studied biomarker of tumor neovascularization for its crucial significance in angiogenesis and vasculogenesis [41]. Through binding to tyrosine kinases receptors, the VEGF signaling pathways play an important role in a variety of physiological and pathological processes. In the process of tumorigenesis, the transcription of several hypoxia-related genes induces the expression of VEGF, mainly via VEGFR-2, to activate angiogenesis [42]. High levels of VEGF expression are linked to endothelial barrier disruption in pathological tumor conditions, promoting cancer distant metastasis [43, 44]. Furthermore, VEGF is involved in regulating the immune response of tumors. A variety of innate immune cells have been reported to secrete VEGF in the tumor microenvironment to reduce the immune response of immune cells to tumor tissue [45–47]. The upregulated VEGF expression was also reported to actively participate in tumor escape from immune surveillance by suppressing the proliferation of T-cells and increasing the exhaustion of T-cells [48, 49].

Recently, it has been implicated that high expression of VEGF mediates metastasis and progression in many malignancies [7–9]. Several meta-analyses have previously assessed the clinical significance of VEGF expression in patients with osteosarcoma [12–14]. Nevertheless, Han et al. focused on the part of the clinicopathological characteristics of VEGF [12]. Researches on the prognostic effect of VEGF expression had inconsistent results and did not pay attention to the quality evaluation, heterogeneity, and sensitivity analysis [13, 14]. Moreover, these researches were published 5 years ago. Limited to the relatively small number of studies, the conclusion was not robust, and some crucial clinicopathological features were not evaluated. Here, we conducted a comprehensive literature search to combine all relevant studies related to VEGF expression's prognostic and clinicopathological value.

In the present meta-analysis study, we pooled 22 studies on VEGF expression in the prognosis or clinicopathology of osteosarcoma patients. In terms of survival data, our findings revealed that overexpression of VEGF was associated with poor overall survival and disease-free survival. The analyses did not find significant heterogeneity or obvious publication bias, and sensitivity analysis showed our results were robust and reliable. Therefore, we supported the hypothesis that elevated VEGF expression predicted poor DFS. In terms of clinicopathological characteristics, similar to previous reports, VEGF overexpression was related to a higher tumor grade and rate of metastasis but not associated with gender, age, tumor location, local recurrence, clinical stage and response to chemotherapy [12]. The results indicated that high levels of VEGF expression predict metastasis and an advanced stage of osteosarcoma. Additionally, previous meta-analyses had not assessed the association between VEGF and MVD. In our study, VEGF overexpression had a marked effect on promoting vascularization in osteosarcoma. However, the results should be interpreted cautiously as only limited studies were included in the analyses, and more related research is needed.

This meta-analysis has some limitations. Firstly, the methods for identifying and evaluating VEGF expression varied among the eligible studies. Although most of these studies applied IHC, the varied antibodies and dilutions utilized may have contributed to heterogeneity. In addition, there were discrepancies in the definition of VEGF positive. The staining methods, the details of the IHC scoring criteria, and cutoff values varied across the included studies. Secondly, the correlation between VEGF expression and some clinicopathological characteristics of osteosarcoma, such as tumor size, were not analyzed in our study due to the insufficient studies using the same criteria of tumor size. Furthermore, when the results of the multivariate survival analysis were reported, the survival data were extracted directly. When not stated in the original articles, the HRs with their corresponding 95% CIs were calculated through the reconstruction of survival curves, which may affect the robustness of the pooled overall survival and disease-free survival. In order to eliminate bias, more precise data extraction methods or better study quality were needed. Lastly, although this study comprised more than 1000 osteosarcoma patients, future studies with larger sample sizes are necessary to further elucidate the association between VEGF and prognosis and clinicopathological characteristics.

## Conclusion

This meta-analysis indicated that elevated VEGF expression was correlated with adverse osteosarcoma clinicopathological features and poor prognosis. Our results suggest that VEGF is a predictive biomarker in patients with osteosarcoma. However, further large-scale, prospective research is required to validate our results.

## Data Availability

The data used to support the findings of this study are available from the corresponding author upon request.

## Abbreviations

VEGF: Vascular endothelial growth factor
HR: Hazard ratios
OS: Overall survival
DFS: Disease-free survival
OR: Odds ratio
CI: Confidence intervals
PRISMA: Preferred Reporting Items for Systematic Reviews and Meta-Analyses
IHC: Immunochemical staining
MVD: Microvessel density
